# A Blind Spot in the Diagnostic Field: The Challenging Diagnosis of Tumefactive Multiple Sclerosis

**DOI:** 10.1155/2018/6841291

**Published:** 2018-06-27

**Authors:** Ramy Mando, Emile Muallem, Shaiva G. Meka, Ramona Berghea

**Affiliations:** ^1^Department of Internal Medicine, William Beaumont Hospital, Royal Oak, MI, USA; ^2^Oakland University William Beaumont School of Medicine, Rochester, MI, USA

## Abstract

Tumefactive Multiple Sclerosis (TMS) is a rare variant with 1 per 1000 cases of MS and 3 per million cases per year. TMS can mimic clinical and radiological features of a neoplasm, infarction, or abscess and therefore can be diagnostically challenging for clinicians. We present a clinical scenario of a patient presenting with left homonymous hemianopia with atypical radiological features initially thought to be more consistent with neoplasm or infraction. Ultimately, biopsy was done which led to the diagnosis of tumefactive multiple sclerosis.

## 1. Introduction

Multiple Sclerosis (MS) is a chronic, inflammatory demyelinating disease of the central nervous system, with a relapsing and remitting quality [1]. Tumefactive Multiple Sclerosis (TMS) is characterized by a plaque size ≥ 2 cm with mass effect, edema, or ring enhancement on magnetic resonance (MR) imaging [2]. It is a rare variant with 1 per 1000 cases of MS and prevalence of 3 cases per million inhabitants per year [3, 4]. TMS can mimic the clinical and radiological features of a neoplasm, cerebral abscess, or inflammatory process and can be diagnostically challenging for clinicians. It is often misdiagnosed, and while neuroimaging may narrow the differential, a biopsy may be needed to make the definitive diagnosis [5, 6]. TMS is characterized by well-demarcated hypointense lesions on computed tomography (CT) and is hyperdense on T2 and relatively hypodense on TI. Ring enhancement with gadolinium has been shown to be characteristic of a tumefactive demyelinating lesions [7, 8].

## 2. Case Description

**Figure 1:**
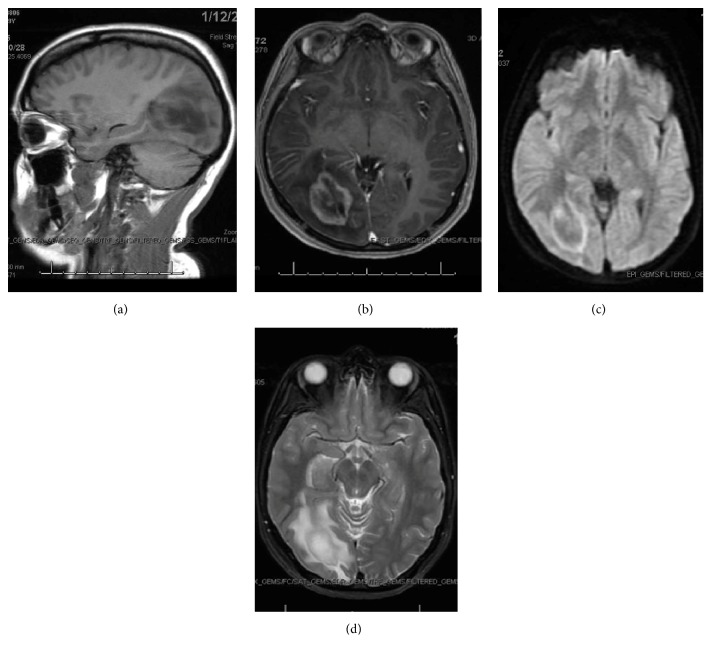
(a) MRI sagittal view T1 FLAIR image. (b) MRI axial T1 BRAVO image. (c) MRI axial DWI image. (d) Axial T2 FSE image. MRI Interpretation: Restricted diffusion in the right occipital lobe. There is compression of the midportion of the occipital horn of the right lateral ventricle. There is enhancement of a presumed mass surrounding the occipital horn of the right lateral ventricle with adjacent vasogenic edema. The possibility that this represents a mass of the right occipital lobe is considered. There is mass effect on the right cerebral hemisphere with effacement of the cortical sulci.

A 29-year-old female with no medical history presented to the hospital for acute left-sided vision loss. Associated symptoms included photophobia, floaters, and bifrontal headache. On examination, she had left homonymous hemianopia, but no other neurologic deficits. Computed tomography revealed acute ischemia involving the right parieto-occipital lobe with vasogenic edema. Magnetic resonance imaging (Figure 1) revealed a mass in the aforementioned region. Initial differential was ischemia versus neoplasm. She was started on dexamethasone and underwent brain biopsy. Pathology revealed white matter infiltration by macrophages intermixed with reactive astrocytes with loss of myelin in the white matter. Myelin was seen within the macrophages. Axons were preserved. Overall the findings were consistent with active demyelination with no findings to suggest neoplasm, most consistent with tumefactive multiple sclerosis. Cerebral spinal fluid (CSF) analysis supported the diagnosis with elevated immunoglobulin G, immunoglobulin G/albumin ratio, and immunoglobulin G index. CSF analysis for oligoclonal bands which is positive in up to 30% of patients with TMS had a negative result in our patient [9]. Despite this, given the patient's clinical presentation and definitive findings on biopsy she was diagnosed with tumefactive multiple sclerosis. She received one gram of intravenous methylprednisolone daily for a total of five days and was then transitioned to prednisone taper. She was discharged with stable neurologic status to inpatient rehabilitation with plans to start immunomodulatory therapy as an outpatient. A four-month chart review of the patient's record revealed that she is doing well. She is currently completing speech therapy with goals to improve mild language deficits including auditory comprehension and integration, verbal expression, and thought organization.

## 3. Discussion

This is a unique case of TMS manifesting as homonymous hemianopia. Studies have shown only 10% of patients with TMS present with visual deficits. Diagnosis of TMS is difficult but should be strongly considered in patients with neurologic deficits and supportive imaging findings. These include mass greater than 2 centimeters, vasogenic edema, ring enhancement, restricted diffusion, and T2 hypointensity. Typically, patients follow a relapsing-remitting course although studies have shown that a mass greater than 5 centimeters, as seen in our patient, is a poor prognostic factor.

In a large study of 168 patients with biopsy confirmed diagnosis of MS, radiographical and clinical information was gathered and patient characteristics, clinical course, MRI findings, and prognosis were evaluated. The ratio of female: male was 1.2:1, median age of onset was 37 years, duration between symptom onset to biopsy was 7.1 weeks, and total disease duration was 3.9 years. From clinical course prior to biopsy, this was first neurological event in 61% of patients, relapse remitting presentation in 29%, and progressive in 4% [3]. Our patient was below the average age at 29 years, and clinical course from symptom onset to biopsy was 2.1 weeks for our patient. At follow-up, 70% of patients developed definite multiple sclerosis, while 14% had an isolated demyelinating disease. Tumefactive features and clinical outcomes were analyzed and lesion size did not correlate with gender, age of onset or biopsy, or clinical course and diagnosis prior to biopsy [3]. However, lesions found to be greater than 5 cm were found to have a slightly higher expanded disability status scale (EDSS). Also this study it is noted that 10% of the patients sampled had a visual field defect, which was found in our patient. Treatment options for tumefactive MS are similar to prototypic MS, with corticosteroids being effective, and studies show promise with Natalizumab as an effective treatment for relapse remitting MS and one case report of plasma exchange therapy being effective in a patient who had corticosteroid resistant tumefactive multiple sclerosis [10, 11]. Fingolimod is a sphingosine 1-phosphate receptor modulator that helps prevent recirculation of lymphocytes. It has been shown to decrease relapses and slow the progression of disability associated with nontumefactive MS. Several case reports have noted the possible conversion to nontumefactive MS into tumefactive MS with the use of this medication and although our patient did not have any exposure to this medication, its association should be noted [12]. Lastly, the absence of oligoclonal bands in patient's diagnosed with TMS has been reported in the literature with definitive diagnosis relying on biopsy in these patients [9, 13, 14].
